# Bodily Emanations

**Published:** 1862-06

**Authors:** 


### BODILY EMANATIONS.

That there are material emanations, distinguishable by the sense of smell, constantly passing from every thing that breathes, is not denied. That every man has a *different,* "scent," is proven by the fact that a dog will follow his master through a crowded street or road, although not in sight, keeping his nose to the ground until he can be seen, when he bounds away with his head upwards, because the eye then assists him. An emanation comes from the negro which it requires no nice olfactory to discover. There are some white persons who will scent a room in five minutes after their entrance, to the extent of really sickening delicate organizations. There are most probably emanations of a still more ethereal character, more spiritual than solid or physical. One unknown person entering a room where there is a promiscuous company, will, without speaking a word, chill the whole party; another will fill it with disgust; while a third will send out a genial influence on every heart. It does not require a very large stretch of the imagination to infer that a combination of the ethereal or spiritual emanations with the more solid or material, may certainly act in such a way as to have a malign influence on a highly wrought or very susceptible organization, especially when brought into so close a contact as that of bed-fellows. It is known the world over, that low typhoid fevers of the most malignant and fatal type are caused by human emanations, by crowding persons in confined apartments. These things being true, there is wisdom in the universal custom of Germany to have all beds single. Such a thing as a double bed would be considered a disagreeable curiosity in that wide-spreading nation. What custom prevails in this respect when the "fatherland" is left, is not known.

